# A novel exfoliated manganese phosphoselenide as a high-performance anode material for lithium ions storage

**DOI:** 10.3389/fchem.2022.949979

**Published:** 2022-09-29

**Authors:** Hailin Shen, Wei Zhang, Yuheng Zhang, Wei Wang, Min Wang, Tianyu Liu

**Affiliations:** School of Chemical Engineering and Materials, Changzhou Institute of Technology, Changzhou, China

**Keywords:** manganese phosphoselenide, exfoliation, anode, cycling stability, LIBs

## Abstract

Layered manganese phosphoselenide (MnPSe_3_) is expected to be a potential anode for Li ions storage due to it combines the merits of phosphorus with metal selenide. It promotes charge transfer and ensures a high theoretical capacity of up to 746 mA h g^−1^. In this work, a comprehensive study clearly demonstrated that bulk MnPSe_3_ electrode is the inability to maintain the integrity of the structure with severe detectable fracture or pulverization after full lithiation/delithiation, resulting in poor rate capability and cycling stability. Additionally, exfoliated few-layered MnPSe_3_ nanoflakes by the ultrasonic method show enhanced electrical conductivity and resistance to volume expansion. It has a high initial discharge/charge capacity reaching to 524/796 mA h g^−1^ and outstanding cycling stability with charge capacities of 709 mA h g^−1^ after 100 cycles at 0.2 A g^−1^ within the potential window of 0.005–3 V vs. Li^+^/Li. While further improving the cycles, the retention rate was still held at ∼72% after 350 cycles. This work provides new insights into exploiting new novel layered materials, such as MnPSe_3_ as anodes for lithium-ion batteries.

## Introduction

As is known, lithium-ion batteries (LIBs) (Liu et al., 2010; Dunn et al., 2011; Goodenough, 2014) have been utilized in countless commodities, such as mobile phones and electric vehicles. However, its wider applications are impeded owing to limiting materials, so there is still great potential as long as more novel electrode materials are exploited for LIBs.

So far, various metal selenides, such as FeSe_2_ (Kong et al., 2019; Yousaf et al., 2020), MnSe(Li et al., 2016; Liu et al., 2018; Li Z. et al., 2019), and CoSe_2_ (Yu N. et al., 2019; Xu et al., 2020) have been deeply studied as active materials in LIBs. Nevertheless, it presents the challenges of inferior rate capability and rapid capacity loss. Some researchers have also focused on phosphorus-based materials such as black phosphorus (Dahbi et al., 2016; Del Rio Castillo et al., 2018), SiP (Duveau et al., 2016), FeP (Jiang et al., 2017), and GeP (Li W. et al., 2019; Li X. et al., 2019; Fan et al., 2019), which also exhibits the inferior cycling performance. Nevertheless, improving the electrochemical performance of metal sulfides and phosphides in terms of rate capability and cycling stability is an enormous challenge due to the large volume change that can lead to crushing and loss of electrical contact.

Recently, a novel ternary metal phosphide sulfide/selenides (MPX_3_, M is transition metal, such as Mn, Zn, *etc*; X is Se or S (Brec, 1986; Pei et al., 2018; Gusmao et al., 2019; Samal et al., 2021), have been investigated in electrocatalysis (Byvik et al., 1982; Dedkov et al., 2020), hydrogen storage (Cabria and El-Meligi, 2018) and toxicological hazards (Latiff et al., 2018). However, the MPX_3_ empolyed as active materials in LIBs are rarely reported. The layered MPX_3_ owns attractive lithium storage ability for rechargeable ion batteries. Its unique two-dimensional (2D) layered nanostructure, which is composed of weak van der Waals stacking between layers, is deemed to be an ideal framework for fast Li^+^ storage. The layered structure alleviates the volume stress, generates abundant ion diffusion pathways and speedy electron transportation owing to lower energy barrier (Fan et al., 2019; Ding et al., 2020). More importantly, the preferred bandgaps\ of MPX_3_ (1.3–3.5 eV) (Wang et al., 2018) and potential ionic conductivity make MPX_3_ as superior anode electrode materials. Some groups have reported like-MPX_3_, such as MnPS_3_ (Sang et al., 2020), CoPS_3_ (Jana et al., 2020), FePSe_3_ (Xing et al., 2020), NiPS_3_ (Dangol et al., 2018) and SnPSe_3_ (Ren et al., 2020), exhibit a promising performance of lithium/sodium ions storage.

The manganese phosphorous selenide (MnPSe_3_) is one of MPX_3_, isostructural with FePSe_3_. The MnPSe_3_ possesses an interlayer spacing of∼0.32 nm (Li et al., 2014), much larger than the diameter of Li^+^ (∼0.152 nm), providing channels for Li^+^ diffusion in the insertion/extraction process. MnPSe_3_ as anodes also have a high theoretical capacity of 746 mA h g^−1^ by forming Li_3_P and Li_2_Se alloy (Li et al., 2013; Gusmão et al., 2019; Tang et al., 2020). However, the numerous overlapping layers of bulk MnPSe_3_ lead to a decreasing active surface area, slow charge transfer, and even poor resistance to volume expansion in lithiation/delithiation. According to previous studies (Abdelkader and Kinloch, 2016; Chen et al., 2016; Shen et al., 2020), mechanical exfoliation can effectively narrow the size and thickness of bulk materials, causing abundant exposed active sites, highly tunable morphology, reduced diffusion length of charge carriers for Li^+^ and perfect resistance to volume change.

In this work, the lithiation/delithiation processing of bulk MnPSe_3_ as the anode in LIBs has been disclosed by half-cell. It occurs to serious volume expansion/contraction for bulk MnPSe_3_ in lithiation/delithiation, even the tracking MnPSe_3_ electrode is unable to maintain high integrity with serious cracks or pulverization. Thus, we reduced bulk MnPSe_3_ to a few layered MnPSe_3_ nanoflakes by mechanical exfoliation and comprehensively compared the storage Li^+^ performances of exfoliated MnPSe_3_ to that by grinding as LIBs anodes. Moreover, the exfoliated MnPSe_3_ electrode shows an initial discharge/charge capacity of 524/796 mA h g^−1^, and a retention rate of 88% and 72% after 100 and 350 cycles, respectively at 0.2 A g^−1^. The improved resistance to expansion and pulverization and rapid reaction kinetics indicate the exfoliated MnPSe_3_ is able to achieve superb cyclic stability. Ultimately, exfoliated MnPSe_3_ is considered a great dynamism and potential anode material with predominant performance in LIBs.

## Experimental section

### Synthesis of bulk and exfoliated MnPSe_3_

All chemicals are available without further treatment. The bulk MnPSe_3_ is prepared by grinding in the agate mortar for about 30 min. The thinner and smaller MnPSe_3_ nanoflakes continue to be processed by ultrasonic exfoliation (Zhang et al., 2016). These bulk particles (100 mg) are reduced to thinner by ultrasonic (1000 W, 4 h) in N-Methyl pyrrolidone (NMP) solvent (150 ml) and centrifugation (3,000 rpm for 20 min) to remove large particles. Then the small-size nanoflakes were obtained by washing and drying in a vacuum oven.

### Preparation of MnPSe_3_ electrodes and the coin-type half-cell for LIBs

The slurry of the MnPSe_3_ electrode was prepared by mixing 70 wt% MnPSe_3_ nanoflakes and 20 wt% carbon nanotubes (CNTs) and 10 wt% carboxymethyl cellulose (CMC), which was spread evenly on a copper foil (load of 1.5–2.0 mg cm^−1^), then the obtained products were cut into a disc (diameter of 10 mm) and dried at 70°C for about 12 h under vacuum. The surface morphology of MnPSe_3_/CNT/CMC electrode is shown in Supplementary Figure S1, exhibiting the MnPSe_3_ nanoflakes embedded in the uniform carbon nanotubes matrix. The carbon nanotubes are able to promote a quick electron/ion transfer and alleviate volume stress. Lithium metal foil, polypropylene (PP), and MnPSe_3_/CNT/CMC electrode sequentially were put into the CR2032 cell case for assembling sequentially in the glove box. The electrolyte is composed of 1 M LiPF_6_ dissolved in EC/DMC/DEC (1/1/1 v/v/v) mixed solution.

### Electrochemical measurements of MnPSe_3_ anode

The galvanostatic charge/discharge, rate performance, and cycle performance of half-cell were performed in the volt range from 0.005 to 3 V (vs. Li^+^/Li). Cyclic voltammograms (CVs) were tested using an electrochemical working station in the voltage range of 0.005–3.0 V (vs. Li^+^/Li) at 0.05 mV s^−1^. Electrochemical impedance spectroscopy (EIS) was conducted in a frequency range of 10 K to 0.1 HZ in the same test system.

### Material test and characterization instruments

Battery testing system (a Land CT 2001A, WuHan, China). Electrochemical working station (a 1,400 Cell Test system, Solartron, China). X-ray diffractometer (XRD-Bruker D2, Cu K radiation, *λ* = 1.5418 Å). A field-emission scanning electron microscopy (SEM, Hitachi-S4800). High-resolution transmission electron microscopy (TEM, JEM 2100, JEOL, Japan, 200 kV). X-ray photoelectron spectroscopy (XPS, ThermoFisher EscaLab 250Xi).

## Results and discussion

**FIGURE 1:**
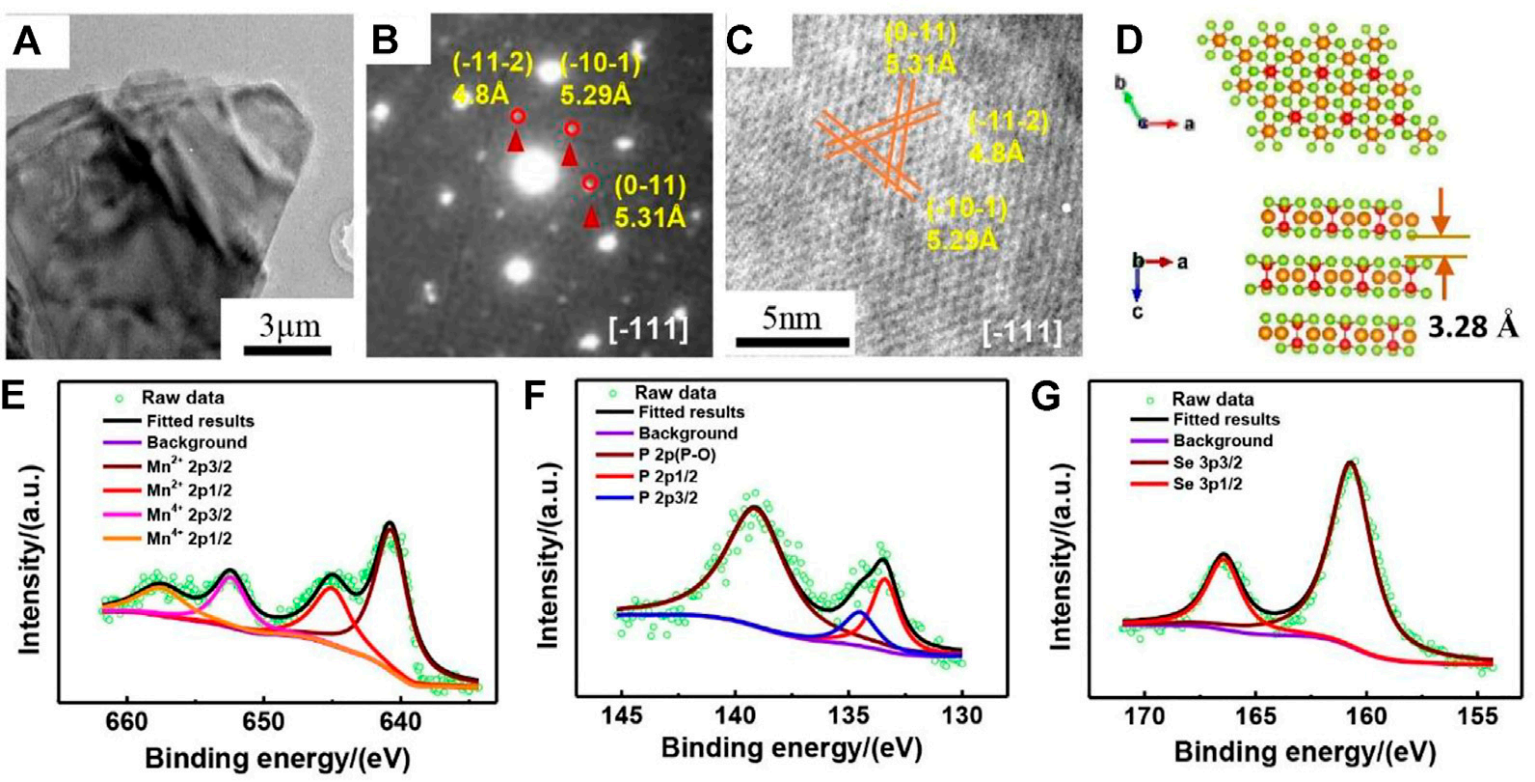
The purity, structure, and composition of MnPSe_3_ nanoflake. **(A)** The typical TEM image of layered MnPSe_3_. **(B)** The selected area electron diffraction (SAED) pattern of MnPSe_3_ nanoflake view from [-111]. **(C)** The high-resolution TEM (HRTEM) image of MnPSe_3_ was viewed along the same direction corresponding to the SAED. **(D)** The crystal structure schematic of MnPSe_3_ along the b and c axes, respectively. **(E–G)** The high-resolution XPS shows spectra of the Mn, P, and Se elements, respectively.

As clearly displayed by the low-magnification TEM in Figure 1A. MnPSe_3_ exhibits an ultrathin and transparent lamellar appearance with several micrometers in plane and nanometers in thickness. The typical SAED pattern of MnPSe_3_ show the diffraction spots of (-11-2), (-10-1), (0-11) plane with corresponding d-spacings of 4.8, 5.29, and 5.31 Å, respectively viewed along [-111] in Figure 1B, which is commensurate to HRTEM along [-111] in Figure 1C. It indicates that the as-prepared MnPSe_3_ possesses high crystallinity and phase purity.

The schematic images of layered MnPSe_3_ viewed from the b and c axes are shown in Figure 1D. MnPSe_3_ belongs to a hexagonal with the lattice parameters of a = 6.387 Å, b = 6.387 Å, c = 19.996Å; and the angle α, β is 90°, γ is 107.35°. As depicted in Figure 1A, a single layer is composed of the Mn atom’s central shell and the other two shells in PS_3_ units. The selenide atoms are located on the two external surfaces of a MnPSe_3_ layer (Li et al., 2014; Pei et al., 2018). Moreover, the spacing of two adjacent MnPSe_3_ layers is 3.2 Å by Van der Waals (Li et al., 2014), which provides channels and buffers volume expansion/contraction for Li^+^ insertion/extraction.

The chemical compositions of MnPSe_3_ nanoflakes were further investigated by XPS in Figures 1E–G. As revealed by the Mn 2p spectrum, the high-resolution Mn 2p profile can be mainly fitted at 640.7 eV (2p3/2) and 651.3 eV (2p1/2), ascribed to the binding energy of Mn^2+^, while the peaks at 642.3 eV (2p3/2) and 657.6 eV (2p1/2) indicate the presence of Mn^4+^ (Sang et al., 2020). The narrowly scanned XPS spectrum of P 2p can be contributed to double peaks at 134.5 and 133.4 eV, which are in line with the P 2p3/2 and P 2p1/2, respectively (Edison et al., 2018; Fan et al., 2019). Additionally, there are only a pair of peaks at 166.4 eV (2p3/2) and 160.7 eV (2p1/2) for the Se 2p (Gusmão et al., 2017; Dedkov et al., 2020), indicating only one form of selenium existed in the MnPSe_3_, in agreement with aforementioned results.

**FIGURE 2:**
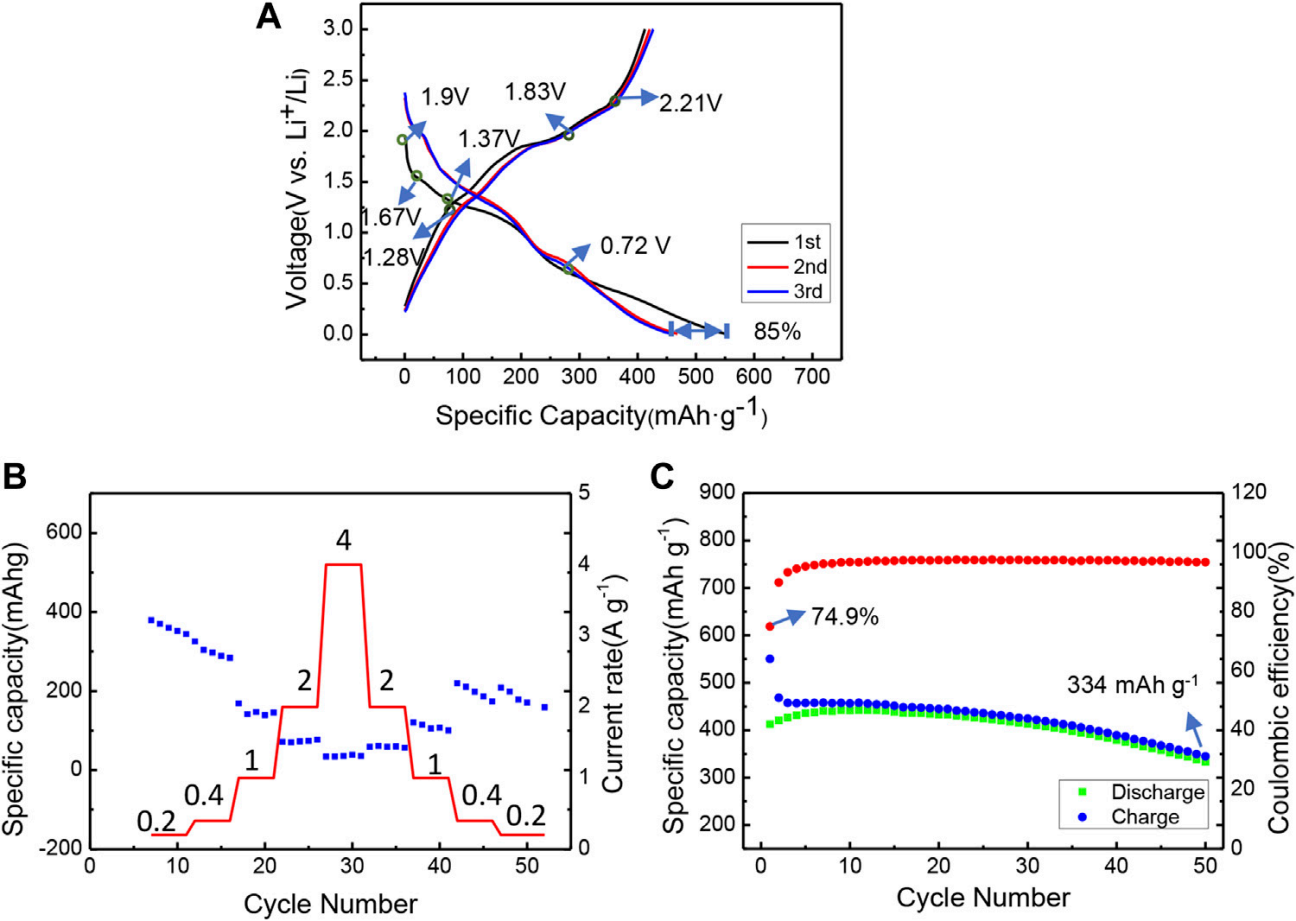
Electrochemical performance of bulk MnPSe_3_ within the window 0.005–3 V (vs. Li^+^/Li). **(A)** The initial three discharging/charging curves of MnPSe_3_ electrode at 0.2 A g^−1^. **(B)** The rate performance at different current densities from 0.2 to 4 A g^−1^. **(C)** The cycling performance of MnPSe_3_ electrode at 0.2 A g^−1^.

Electrochemical behaviors of bulk MnPSe_3_ have been tested in Figure 2. There are mainly four obvious plateau regions at 2.0–1.8 V, 1.75–1.58 V, 1.53–1.15 V, and 0.75–0.35 V in the first discharge curve, while the charge profile also shows three corresponding three plateaus at 2.07–2.34 V, 1.71–1.94 V, and 1.1–1.38 V, respectively in Figure 2A. Significantly, the bulk MnPSe_3_ electrode illustrates the rate capabilities of 0.2, 0.4, 1, 2, and 4 A g^−1^in Figure 2B. With the increase of current density, the specific capacities decay obviously for the MnPSe_3_ electrode. When the current density reaches up to 4 A g^−1^, the reversible capacity of 35 mA h g^−1^ is just left. The bulk MnPSe_3_ delivers a first 412/550 mA h g^−1^ discharge/charge capacity with ∼75% initial Coulombic efficiency in a potential of 0.005–3 V at 0.2 A g^−1^, and an extremely obvious downward trend with a retained capacity of 344 mA h g^−1^ after 50 cycles in Figure 2C. It indicates the cycling durability for the bulk electrode is really poor. The bulk MnPSe_3_ electrode possesses an inferior rate capability and more unstable cycling performance.

**FIGURE 3:**
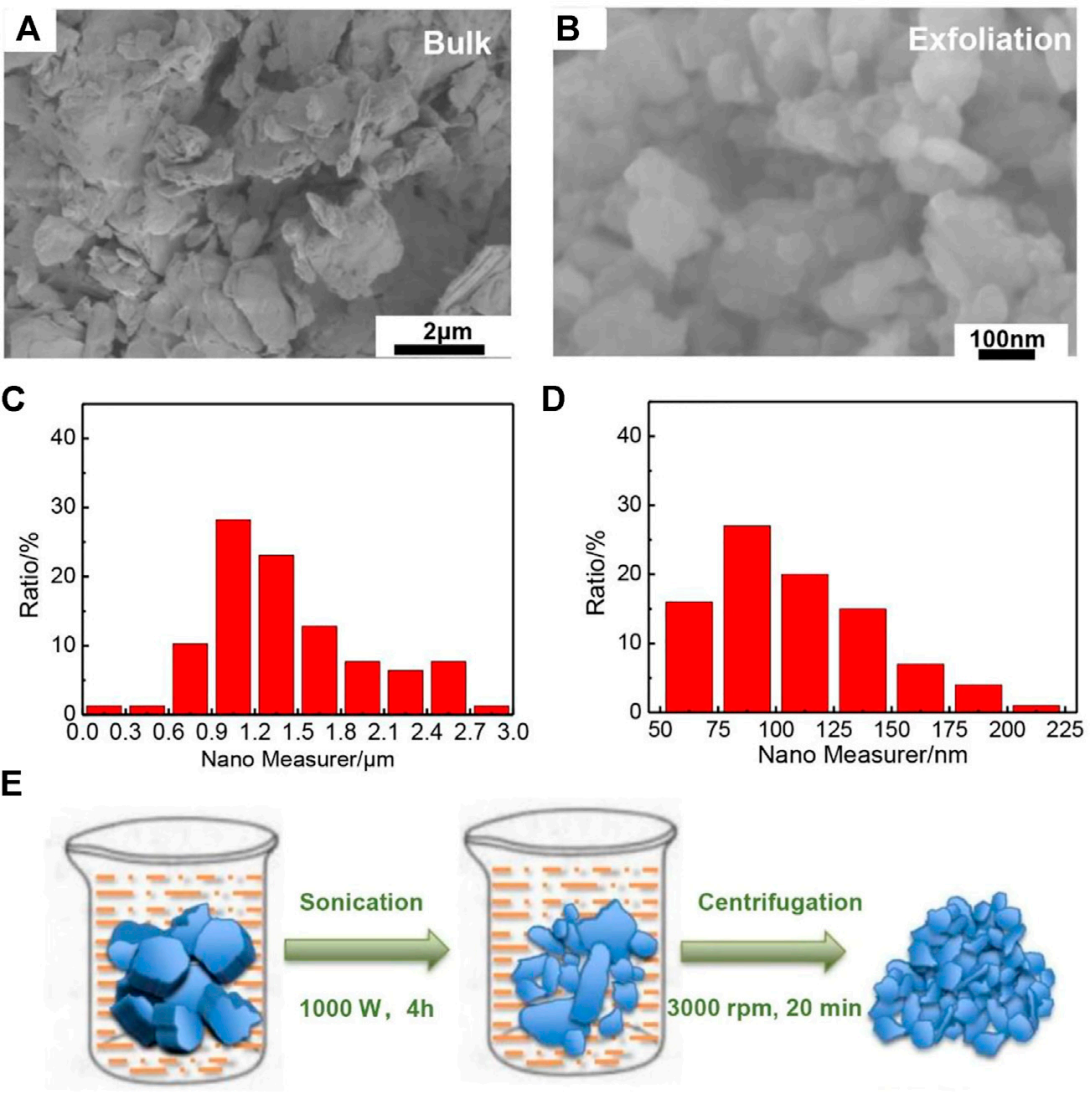
Comparison of the morphology for bulk and exfoliated MnPSe_3_. **(A,B)** The SEM images of MnPSe_3_ nanoflakes by hand grinding and exfoliation. **(C,D)** The size distribution of bulk and exfoliated MnPSe_3_ nanoflakes corresponds to Figures 3A,B. **(E)** The processing illustration of MnPSe_3_ nanoflakes was obtained by sonication-assisted exfoliation.

In order to further improve the Li^+^ storage performance of MnPSe_3_, the bulk MnPSe_3_ was refined to nanoflakes by mechanical exfoliation. We compare the morphology between bulk MnPSe_3_ and exfoliated MnPSe_3_ by SEM in Figures 3A, B; Supplementary Figure S2. Compared to bulk MnPSe_3_, exfoliated MnPSe_3_ nanoflakes display a smaller and more uniform size. As shown in Figures 3C,D, the size distribution of MnPSe_3_ nanoflakes was measured by particle size analysis. The size of bulk MnPSe_3_ by hand grinding reaches ∼1.2 μm. However, exfoliated MnPSe_3_ nanoflakes have been largely narrowed to tens of nanometers. Moreover, exfoliated MnPSe_3_ electrode exhibits more remarkable electrical conductivity than bulk MnPSe_3_ in Supplementary Figure S3. According to reported articles (Chen et al., 2016; Dangol et al., 2018; Yu Z. et al., 2019), reducing the size and thinning the thickness of bulk 2D materials can effectively improve abundant exposed active sites and resistance to expansion/shrinkage and shortened diffusion length of charge carriers for Li ions and in the process of Li^+^ insertion and extraction. In addition, as shown in Figure 3E, MnPSe_3_ nanoflakes were prepared by two-step method. The liquid-phase ultrasonic exfoliation does not involve in phase transformation and any new phases formation. In addition, this method achieves controllable size nanoflakes and high repeatability. The obtained MnPSe_3_ nanoflakes exhibits further enhancement on fast chargeability and long cyclability of Li^+^ storage. Firstly, bulk MnPSe_3_ were crumbled roughly by ultrasonic stripping. Secondly, smaller MnPSe_3_ nanoflakes effectively were separated by fractional centrifugation.

**FIGURE 4:**
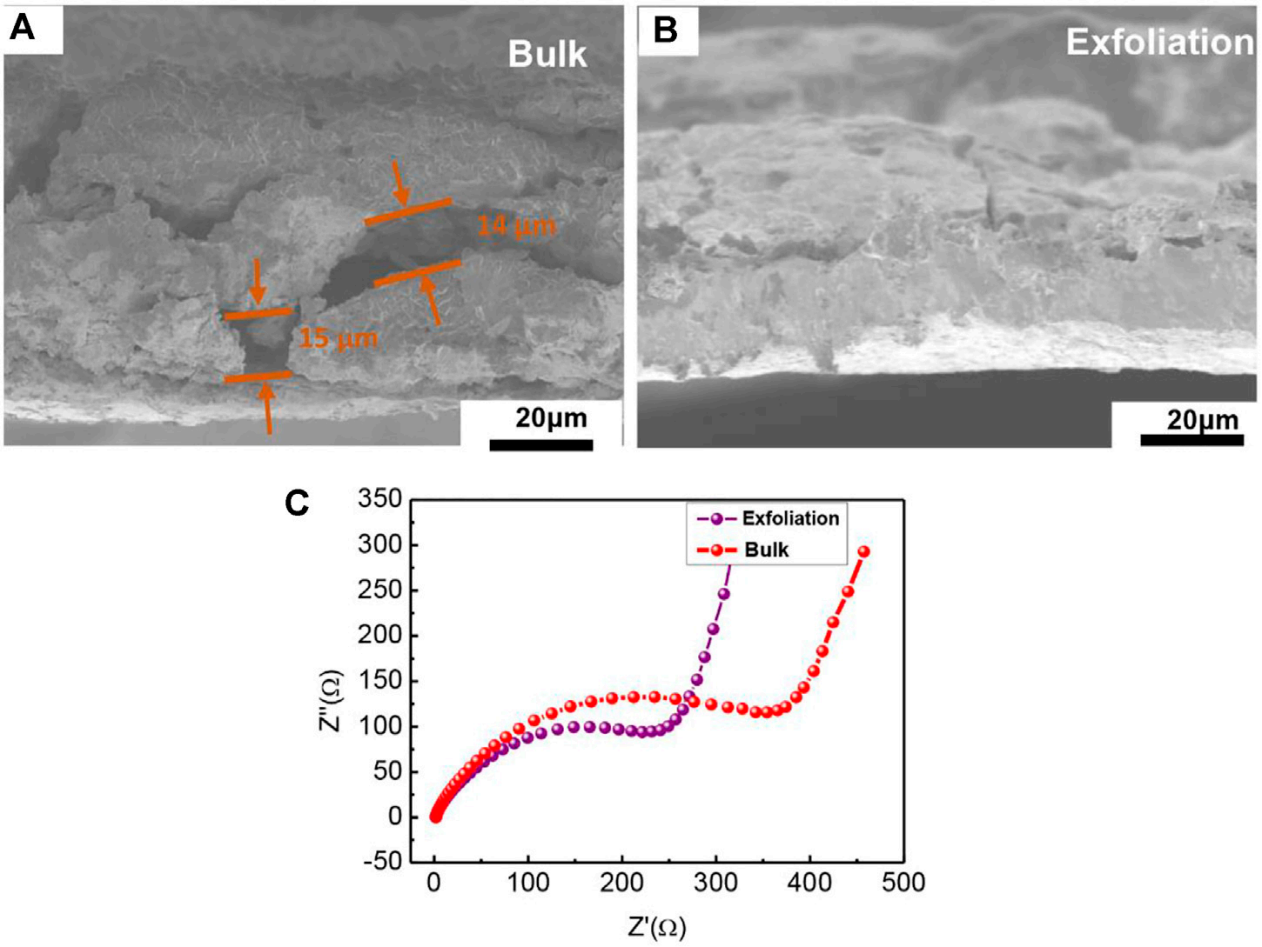
The performance analysis of MnPSe_3_ electrode. **(A,B)** The side SEM of MnPSe_3_ electrode before and after exfoliation in 100 charge/discharge cycles. **(C)** The impedance of MnPSe_3_ electrode in 100 charge/discharge cycles before and after exfoliation, respectively.

Furthermore, we examined the discrepancy of the bulk and exfoliation MnPSe^3^ in morphology and EIS, respectively in Figure 4. The side surface of exfoliated MnPSe^3^/CNT/CMC electrode shows serious cracking, reaching ∼15 μm due to severe volume expansion/shrinkage after full lithiation/delithiation in Figures 4A,B, which is a key cause of rapid failure for bulk MnPSe^3^ electrode. While it was found that exfoliated MnPSe^3^ remained integrity after 100 cycles. This clearly further demonstrates that exfoliated MnPSe^3^ electrodes resist severe volume expansion owing to possessing excellent mechanical robustness. Interestingly, after 100 charge/discharge cycles, the thinner and smaller layered MnPSe^3^ electrode obtains a lower transfer resistance than bulk MnPSe^3^ owing to the contact separation of electrode internal components in Figure 4C, even falling from the current collector, resulting in the decreasing of electrical conductivity and ion transport properties. Thus, the exfoliated layered MnPSe^3^ electrode facilitates Li^+^ extraction from the insertion region.

**FIGURE 5:**
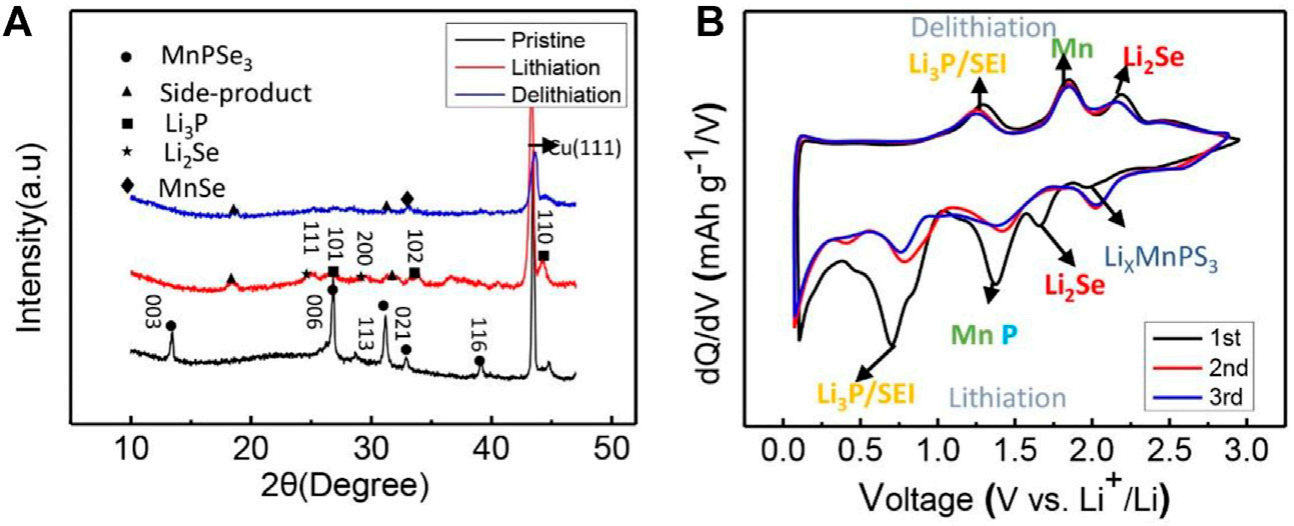
Phase characterization of MnPSe_3_ electrode in lithiation/delithiation. **(A)** The *ex-situ* XRD patterns of MnPSe_3_ electrodes for the first lithiation/delithiation. **(B)** The differential capacity curves of the MnPSe_3_ electrode in the initial three cycles.

To further confirm the phase transformation of MnPSe_3_ in lithiation/delithiation, X-Ray Diffraction (XRD) has also been performed on the MnPSe_3_ electrode in Figure 5A. The pristine MnPSe_3_ electrode exhibits the obvious crystallographic orientations of (003), (006), and (113), and no detectable impurities were found. The *ex-situ* XRD of the MnPSe_3_ anode presents Li_3_P peaks at about 26.6°, 33.8°, and 44.3° (Kim and Cho, 2009), and Li_2_Se peaks at about 25.1° and 22.6° after the first full lithiation (Liu et al., 2020), which further verify single-crystal MnPSe_3_ is entirely alloyed to Li_3_P and Li_2_Se phase. While upon full delithiation, it presents a new peak at 33.1°, which is caused by the MnSe phase (Xue and Fu, 2007). The marked peaks located at other degrees originate from electrolyte decomposition on the surface of the MnPSe_3_ electrode, which is in good agreement with reported results about like-MPX_3_. Significantly, the differential capacity profiles display excellent reversibility in a redox reaction and agree well with the *ex-situ* XRD analysis, which also presents the reduction peak at 1.95, 1.66, 1.30, and 0.6 V, could correspond to the alloying reactions of Li_x_MnPSe, Li_2_Se/P/Mn Li_3_P, and SEI, respectively. Considering the analysis above, phase transformation in first lithiation/delithiation could be summarized as follows:

After First lithiation:

$${MnPSe}_{3}+9{Li}^{+}+9e^{-}\to {Li}_{3}P+Mn+3{Li}_{2}Se$$ (1)

After First delithiation:

$${Li}_{3}P+Mn+{Li}_{2}Se\to MnSe+P+5{Li}^{+}+5e^{-}$$ (2)

**FIGURE 6:**
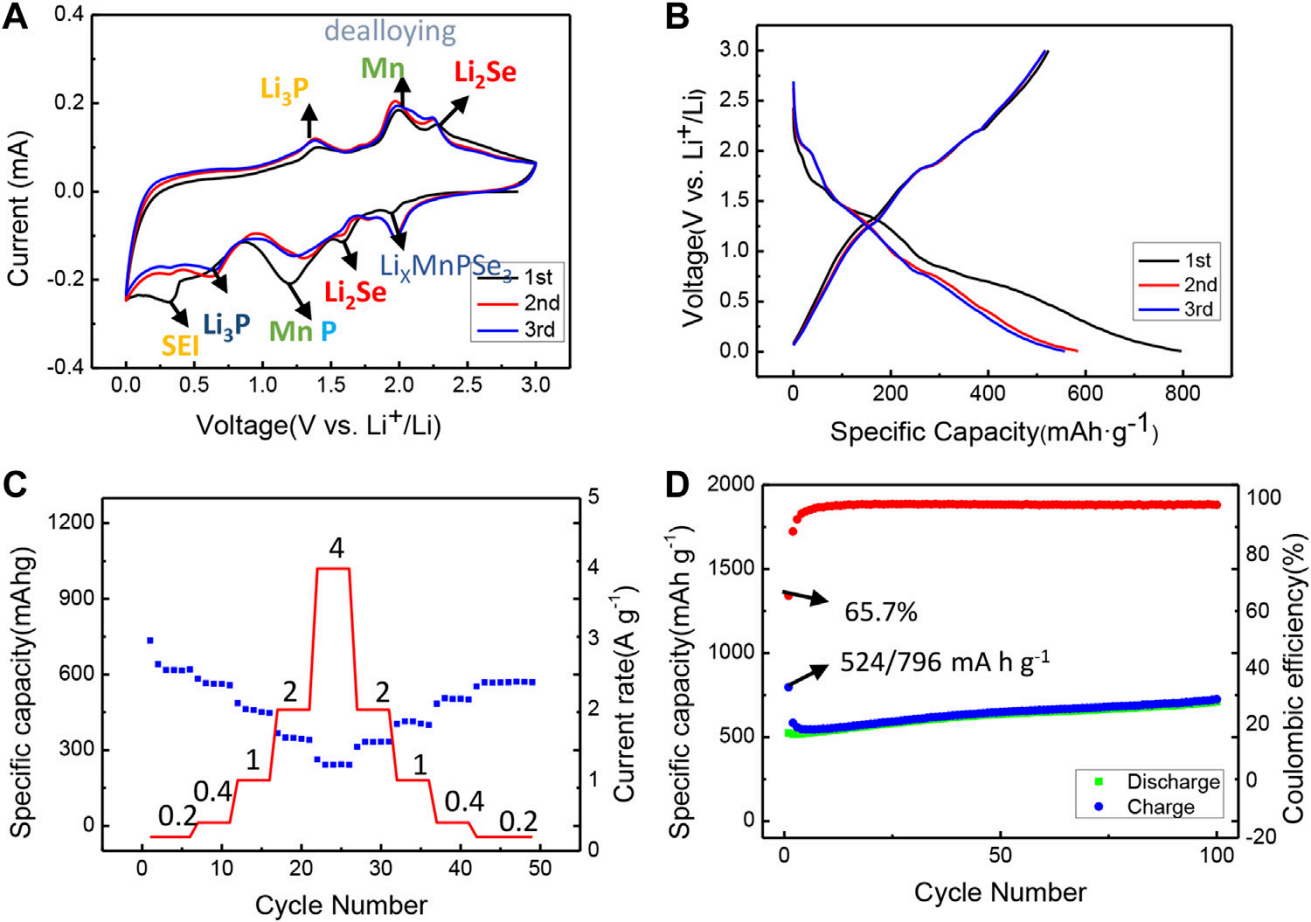
Electrochemical performance of exfoliated MnPSe_3_ as anode in Li ions half-cell. **(A)** The CVs of the initial three cycles in the voltage window of 0.005–3 V at 0.05 mV s^−1^. **(B)** Galvanostatic discharge/charge profiles for the first three cycles in the voltage window of 0.005–3 V (vs. Li^+^/Li) at 0.2 A g^−1^. **(C)** Rate performance of MnPSe_3_ anode within the potential of 0.005–3 V vs. Li^+^/Li at different current densities from 0.2 A g^−1^ to 4 A g^−1^. **(D)** Cycling performance of exfoliated MnPSe_3_ electrode tested within the potential of 0.005–3 V (vs. Li^+^/Li) at 0.2 A g^−1^.

As shown in Figure 6, the exfoliated MnPSe_3_ anode is further utilized in a half-cell. Primarily, the typical cyclic voltammogram (CV) curves of the electrode were illustrated in Figure 6A. The initial cathodic sweep displays four distinct reduction peaks at 1.95 V, 1.6 V, 1.2 V, 0.6 V, and 0.35 V, indicating the lithiation/delithiation process is a multiple-step. The prominent peak located at 1.95 V is matched to Li_*x*_MnPSe_3_. The peaks centered at 1.6 V and 1.2 V are associated with the formation of Li_2_Se, P, and Mn. The peak at 0.6 V is related to the generation of Li_3_P. Another weak broad peak located at 0.35 V is attributed to the side reaction (formation of SEI film). In the following anodic sweep. The three strong peaks at 1.38 V, 2.0 V, and 2.27 V are coincident with dealloying of Li_3_P, Li_2_Se, and the formation of MnSe. The result above is similar to CVs profiles for like-MPX_3_ (FePSe_3_, NiPS_3,_ etc) (Sang et al., 2020; Xing et al., 2020; Liu et al., 2021). In addition, the initial three cycles curves exhibit a consistent property of reaction to that of the CV results above in Figure 6B. The charge/discharge curves and CVs in multiple cycles are also nearly overlapped, suggesting the wonderful stability of electrode.

To evaluate the lithium storage properties of the exfoliated MnPSe_3_ at _a_ high rate, the reversible capacities of 616, 562, 458, 348, and 242 mA h g^−1^ were obtained at current densities of 0.2, 0.4, 1, 2, and 4 A g^−1^, respectively in Figure 6C. Moreover, the capacity achieves 331, 412, 502, and 568 mA h g^−1^ with the current density coming back to 2, 1, 0.4, and 0.2 A g^−1^, illustrating the MnPSe_3_ electrode maintains a remarkable rate performance. Compared to bulk MnPSe3, the cycling stability of the exfoliated MnPSe_3_ is also effectively improved. It maintains outstanding cycling stability with capacity retention of 709 mA h g^−1^ after 100 cycles at 0.2 A g^−1^, and capacity retention of 578 mA h g^−1^ after 350 cycles at 0.2 A g^−1^in Figure 6D; Supplementary Figure S4.

## Conclusion

In summary, this work researches on performance improvement of the MnPSe_3_ as the anode of LIBs in detail by ultrasonic exfoliation, revealing an extraordinary ability to resist volume expansion/shrinkage in full lithiation/delithaition, which provides significant evidence for the research of like-MPX_3_. The thinner and smaller MnPSe_3_ shows superior performance to the bulk electrode material. When supplied as the anode of LIBs in half-cell, a splendid reversible capacity of 709 mA h g^−1^ was maintained for the MnPSe_3_ within the potential window of 0.005–3 V vs. Li^+^/Li after 100 cycles at 0.2 A g^−1^. While further improving the cycles, a specific capacity of 578 mA h g^−1^ was still held after 350 cycles, which benefits from the favorable capacitance kinetics, and resist severe volume expansion. Layered MnPSe_3_ as anode materials for LIBs meet the needs of high capacity, rapid charge-discharge, and long cycle.

## Data Availability

The original contributions presented in the study are included in the article/Supplementary Material further inquiries can be directed to the corresponding authors.
