# Supplementary material for: A novel exfoliated manganese phosphoselenide as a high-performance anode material for lithium ions storage
Source: Front Chem. 2022 Sep 29;10:949979. doi: 10.3389/fchem.2022.949979 (PMC9559372; doi:10.3389/fchem.2022.949979)
Supplement: Supplementary file 1 [file DataSheet1.docx]

Supplementary Information for

**A novel exfoliated manganese phospho****[selenide](javascript:;) as a high-performance anode material for lithium ions storage**

Hailin Shen^a^, Wei Zhang^a^, Yuheng Zhang^a^, Wei Wang^a*^, Min Wang^a*^ Tianyu Liu^a*^.

*^a^School of Chemical Engineering and Materials, Changzhou Institute of Technology*

*Changzhou, China;*

*To whom correspondence should be addressed. E-mails:* [*wangwei2017@czu.cn*](mailto:wangwei2017@czu.cn)*(Wei Wang), milladengdai@hotmail.com(Min Wang),* [*liuty@czu.cn*](mailto:liuty@czu.cn)*(Tianyu Liu).*


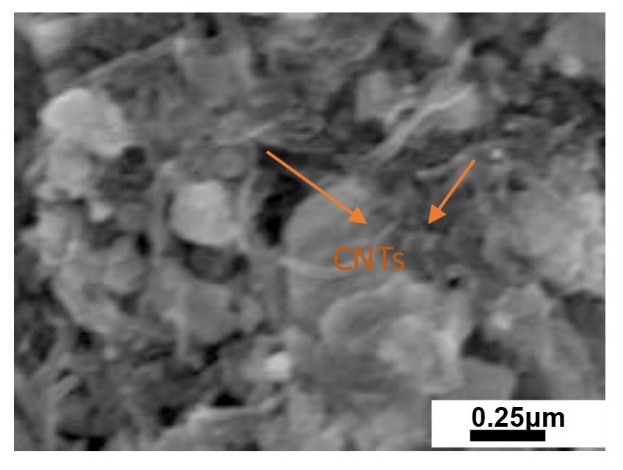


**Figure S1.** SEM images of MnPSe_3_/CNT/CMC electrode.


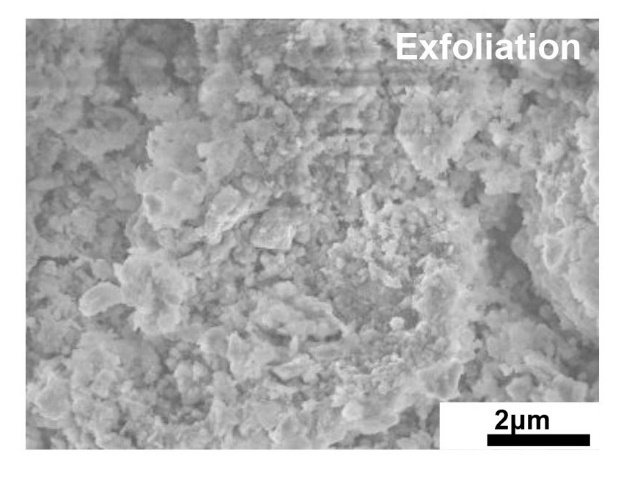


**Figure S2.** SEM images of MnPSe_3_ nanoflakes after exfoliation.


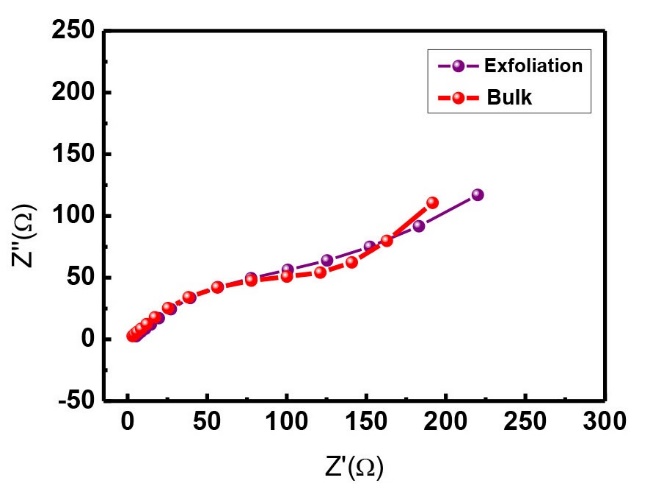


**Figure S3.** The impedance of MnPSe_3_/CNT/CMC electrode before and after exfoliation, respectively.


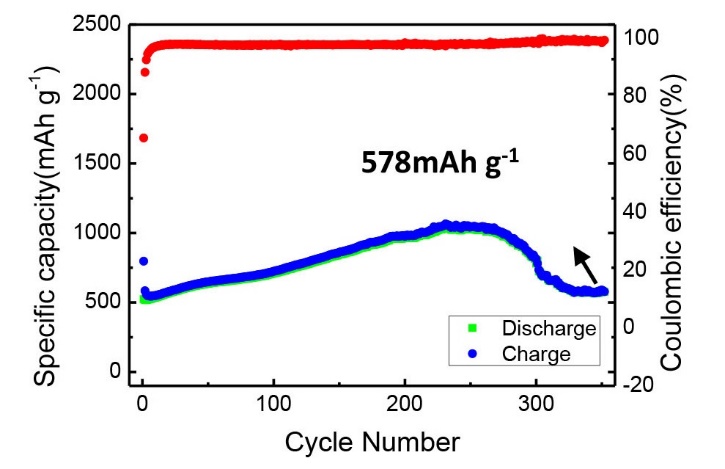


**Figure S4.** Cycling performance MnPSe_3_ electrode by exfoliation after 350 cycles at 0.2 A g^−1^ in the voltage of 0.005-3 V.
